# Commentary on: The Anatomy of a Malpractice Lawsuit

**DOI:** 10.1093/asjof/ojad012

**Published:** 2023-02-03

**Authors:** Caroline A Glicksman

The authors provided a detailed and comprehensive summary of a medical malpractice suit, as well as recommendations for physicians to avoid lawsuits in their practice (Video).^1^ Notably, they discuss the issue of patient dissatisfaction as a common adverse event in lawsuits. Unhappiness may be a subjective measure of poor performance by a patient and often indicates a failure to meet a patient's expectations. Figuring out what a patient understands, imagines, or believes is the key to managing patient expectations from the very beginning of the doctor–patient relationship.

Surgeons should be as accurate, truthful, and as forthcoming as possible when discussing the available courses of action and possible outcomes.^2^ Brutal honesty and open communication about what is possible or realistic from a specific procedure, especially in revisionary surgery, is the best approach in managing expectations. Patients should be reminded that their surgeon can do everything right, and make every right decision, but cannot guarantee a specific outcome.

Patients with unmet expectations often fail to return for follow-up visits and may seek other surgical opinions or legal advice. Being painfully clear with patients about their expectations and helping them understand their specific situations thoroughly, will result in patients receiving better care and being less likely to sue because of a misunderstanding.

## Supplementary Material

ojad012_Supplementary_Data
